# Time to act on headache disorders

**DOI:** 10.1007/s10194-011-0368-7

**Published:** 2011-08-28

**Authors:** Timothy J. Steiner, Lars Jacob Stovner, Tarun Dua, Gretchen L. Birbeck, Rigmor Jensen, Zaza Katsarava, Paolo Martelletti, S. Saxena

**Affiliations:** 1 Department of Clinical Neuroscience, Norwegian National Headache Centre, St Olavs Hospital, Norwegian University of Science and Technology (NTNU), 7006 Trondheim, Norway; 2 Imperial College London, London, UK; 3 World Health Organization, Geneva, Switzerland; 4 Michigan State University, East Lansing, MI USA; 5 Chikankata Health Services, Mazabuka, Zambia; 6 Danish Headache Centre, Glostrup Hospital, University of Copenhagen, Glostrup, Copenhagen, Denmark; 7 University of Essen, Essen, Germany; 8 Sapienza University of Rome, Rome, Italy

Headache is felt, at some time, by nearly everybody, and almost half the world’s adults at any point in time have recent personal experience of one or more headache disorders [1]. In the Global Burden of Disease Study, updated in 2004, migraine on its own accounted for 1.3% of all years of life lost to disability worldwide [2]. Other headache disorders, collectively, may be responsible for a similar burden [1]. Yet, much is unknown about the public-health impact of these disorders. Not only is our view incomplete of the global burden attributable to headache disorders, but also our knowledge of health-care resource allocation to them is scant.

The World Health Organization (WHO) initiated *Project Atlas* with the objective of collecting, compiling and disseminating relevant information on health-care resources in countries. Within *Project Atlas*, information has been gathered for various domains of mental and neurological services and conditions of public-health priority. The *Atlas of Headache Disorders and Resources in the World* *2011* [3], an important addition to this series, presents information on the burden of headache disorders and the resources available to reduce them. The information was acquired by WHO in collaboration with *Lifting The Burden* (an international non-governmental organization in official relation with WHO) as a project within the Global Campaign against Headache [4]. Most of the data were obtained through a questionnaire survey of neurologists, general practitioners and patients’ advocates from 101 countries, representing 86% of the world’s population. Epidemiological data were compiled from published studies through a systematic review [1], and supplemented by data gathered in population-based studies undertaken within the Global Campaign [5].

What were the findings of this first global enquiry into these matters? They were that headache disorders are ubiquitous, prevalent, disabling and, although largely treatable, under-recognized, under-diagnosed and under-treated. Very large numbers of people disabled by headache do not receive effective health care, so that illness that could be relieved is not, and burdens, both individual and societal, persist. The barriers responsible for this vary throughout the world, but poor awareness in the context of limited resources generally—and in health care in particular—is high among them everywhere.

In summary, the findings illuminate worldwide neglect of a major public-health problem, and reveal the inadequacies of responses to it in countries throughout the world. The implications of these findings show the way forward.

## Knowledge gaps must be filled

Despite that headache disorders impose such disability worldwide, knowledge to inform policy is still incomplete. Further well-conducted epidemiological studies, incorporating population-based measures of individual and societal burdens, are needed in many countries, and especially those that are resource-poor.

## Health care for headache disorders must be improved

Worldwide, about 50% of people with headache are primarily self-treating, and not in contact with any health professionals. This is reasonable: much tension-type headache and some migraine manifests only as infrequent and/or mild attacks. On the other hand, if diagnosis rate reflects quality and reach of headache services, which is likely, there is much room for improvement in all regions. At best the diagnosis rate is 40%, meaning that 60% of people with headache disorders are not properly diagnosed. For medication-overuse headache, a high cause of disability that is both preventable and remediable, the diagnosis rate is 10%. Since this disorder is unlikely to resolve without medical care, this is a failure of health care that has important adverse health and economic consequences.

Guidelines for diagnosis and treatment will support better management, particularly by non-experts in primary care. In many countries that lack them—which are, especially, low-income countries —there is a low-cost opportunity for substantial service improvement.

Again worldwide, the *Atlas* reveals high rates of investigations performed to support diagnosis. This is not expected, since headache disorders mostly do not require investigations, either for diagnosis or assessment. Substantial reductions are possible, with resource savings that can be channelled into better medical care.

Assessment of impact of headache is part of management, needed especially where resources are limited in order to direct them efficiently. Existing assessment instruments are easy to use, but are employed in only a quarter of responding countries. There is a large and low-cost opportunity for improvement through their wider usage, particularly in resource-poor countries.

Many effective drugs exist for headache disorders, but countries in all income categories identify lack of access to them as a barrier to best management. In particular, triptans should be used in preference to ergotamine, which not only has inferior efficacy but also raises concern over toxicity, accumulation and overuse potential [6]. For these reasons, triptans need to be more widely available.

Reimbursement of drug costs is, for many people, the key to better access to drugs. Reimbursement has obvious societal cost implications, but these must be considered in full. Given the cost-effectiveness of most drugs for headache, policies of wider reimbursement appear sensible from a societal perspective.

## Headache services must be organized

The headache disorders that cause most population ill-health are migraine, tension-type headache and medication-overuse headache. It is primarily for these disorders that headache services throughout the world must cater.

Headache services need to be delivered countrywide, efficiently and equitably to a very large number of people who stand to benefit from them. Organization of services to achieve this is clearly a challenge, perhaps with no single, complete and universally appropriate solution, but always their basis must be in primary care. This is where the great majority of people with headache are and should be managed. The proportion of 10% currently seen by specialists is far too great: specialist services are required by and should be reserved for only the very small minority who need them.

A strong efficiency-based argument therefore exists for expanding primary-care management of headache disorders, and this is particularly so in countries where health-service reforms are, generally, shifting priority towards primary care.

## Education is central to remedial action

Lack of education was seen as the key issue impeding good management of headache, and better professional education ranked far above all other proposals for change (75% of the countries that responded to the enquiry). Accordance of low priority to headache disorders means they are given little educational emphasis in medical training which translates later into ineffective management and poor outcomes. Change can only follow recognition of the amount of ill-health these disorders cause, and reassessment of priority accordingly.

Education is required at multiple levels. Most importantly, health-care providers need better knowledge of how to diagnose and treat the small number of headache disorders that are of public-health importance. This better knowledge will improve usage of available treatments, produce better outcomes, avoid wastage and reduce overall costs.

Because most headache should be treated in primary care, emphasis should first be on undergraduate training, in medical schools, requiring changes to the undergraduate curriculum. At present, worldwide, just four hours are committed to headache disorders in courses lasting 4–6 years. Second, it should be on continuing medical education for general practitioners.

As noted earlier, worldwide about 50% of people with headache are primarily self-treating, and not in contact with any health professionals. Therefore, education of people with headache about how to treat their headaches effectively and efficiently is of considerable public-health importance. In better-resourced countries especially, one focus of education should be the avoidance of medication overuse and its consequence of medication-overuse headache, itself a high cause of disability.

## National professional organizations should be supported

National professional headache organizations for headache disorders exist in two-thirds of countries that responded, with a very marked difference between high- and upper middle-income (71–76%) and low-income countries (16%). The true figures may be much lower, as respondents were much more readily identified in countries with such organizations. But where these organizations exist, they have clear roles in promoting education, producing locally relevant management aids, including guidelines, and importing knowledge and international standards through links to international groups. Support for the establishment and maintenance of these organizations appears highly worthwhile.

## Political will is needed

For all of these, if they are to be effective ways forward, there is an urgent need for political recognition that the problem exists, and that it demands remedial action. The *Atlas of Headache Disorders* is intended to have this effect.

Apart from the humanitarian burden of pain and debility and the public ill-health arising from headache, the financial costs of headache disorders to society through lost productivity are enormous [7]—far greater than the health-care expenditure on headache in any country [8]. Investment in well-organized headache services, supported by education, is highly sensible, and may well be cost-saving overall. Governments need to take note.
